# Cerebral Vasospasm after Aneurysmal Subarachnoid Hemorrhage: Mechanism and Therapies

**DOI:** 10.1155/2014/679014

**Published:** 2014-09-08

**Authors:** Chih-Lung Lin, Aaron S. Dumont, John H. Zhang, Mario Zuccarello, Carl Muroi

**Affiliations:** ^1^Department of Neurosurgery, Kaohsiung Medical University Hospital, Kaohsiung 807, Taiwan; ^2^Faculty of Medicine, Graduate Institute of Medicine, College of Medicine, Kaohsiung Medical University, Kaohsiung 807, Taiwan; ^3^Department of Neurosurgery, Tulane University, New Orleans, LA 70112, USA; ^4^Departments of Neurosurgery, Physiology, and Anesthesiology, Loma Linda University School of Medicine, Loma Linda, CA 92354, USA; ^5^Department of Neurosurgery, University of Cincinnati, Cincinnati, OH 45219, USA; ^6^Neurocritical Care Unit, Department of Neurosurgery, University Hospital Zurich, Frauenklinikstrasse 10, 8091 Zurich, Switzerland; ^7^Department of Neurosurgery, Kantonsspital Aarau, Tellstrasse, 5001 Aarau, Switzerland

Although cerebral vasospasm (CV) after aneurysmal subarachnoid hemorrhage (SAH) has been recognized for more than half a century, its pathophysiologic mechanism remains elusive [1]. Delayed CV has classically been considered as the leading and treatable cause of mortality and morbidity in patients following aneurysmal SAH. Despite intensive research efforts, SAH-induced CV remains incompletely understood from both the pathogenic and the therapeutic perspectives. Many pathological processes have been proposed to explain the pathogenesis of delayed CV after SAH, including endothelial damage, smooth muscle contraction, changing in vascular responsiveness, and inflammatory and/or immunological response of the vascular wall [2]. At present, the most important and critical aspects of SAH-induced CV are its failure to consistently respond to treatment and only partial success could be achieved in both experimental models and clinical trials.

For patients with SAH surviving the early phase, secondary ischemia (or delayed cerebral ischemia, DCI) is popularly considered as the leading determinant of poor clinical outcome. Amongst the complications after SAH, CV has been regarded as the major cause of DCI. However, there have been an increasing number of evidences supporting multiple etiologies of DCI other than CV. Although radiographic CV is presented in up to 70% of SAH patients, only 20–30% of all SAH patients suffer from clinically symptomatic CV [3]. Nonetheless, it is now evident that CV alone is inadequate to completely explain DCI following aneurismal rupture [2, 4]. Recent studies on the treatment of CV have failed to solidly support the correlation between angiogram-shown improvement in CV and prognosis. Besides, various drugs proven effective for better functional outcomes have demonstrated their independency of CV reduction. Currently, a multifactorial etiology for DCI has emerged, whereas the role of CV has shifted from the major and most significant determinant to one contributing factor, just like any other factors, to the process. The study of the pathophysiology of DCI has become more broad-minded with several other different mechanisms being actively investigated.

The term “early brain injury” (EBI) was first postulated in 2004, more than 40 years after delayed CV was first described, to explain the acute pathophysiological events occurring within 72 hours of SAH [5, 6]. These events include cerebral autoregulation and blood-brain barrier disruption, activation of inflammatory pathways, excitotoxicity, oxidative stress, and activation of apoptosis [7]. These are direct effects of blood clot in the subarachnoid space and also of transient cerebral ischemia, leading to brain injury not confined to the site of hemorrhage. Many mechanisms of EBI contribute to the pathogenesis of DCI and are hence accountable for the poor outcomes. Causes of DCI have been attributed to the combined effects of delayed CV, activation of proapoptotic pathways, disruption of the blood-brain barrier, arteriolar constriction, thrombosis and dysfunction in microcirculation, and cortical spreading ischemia, all brought about by EBI [2].

Accumulating data have suggested that apoptosis is a key mediator of secondary brain injury after SAH [8]. Approximately, 50% of SAH survivors remain permanently disabled because of cognitive dysfunction and do not return to their previous functions [9]. CV alone could not explain the whole subtle changes in behavior and memory. In this aspect, apoptosis induced by global ischemia should be taken into consideration.

In this special issue, an update review of the mechanism and treatment of CV and DCI after aneurysmal SAH is presented. The roles of mechanisms including microclot formation, downregulation of endothelial nitric oxide synthase, and upregulation of relaxin are discussed. Treatment with progesterone, which attenuates experimental SAH-induced CV by upregulation of endothelial nitric oxide synthase via Akt signaling pathway, is investigated. Besides, a study on Magnesium Lithospermate B, an active extract of salvia miltiorrhiza mediating sGC/cGMP/PKG translocation to reduce CV, is reported. Furthermore, new strategies using 17*β*-estradiol, targeting at several CV-preventing mechanisms, have brought light to the reduction of CV and secondary brain injury after SAH. The treatment and outcome including extracerebral organs damage and long-term complications after aneurysmal and nonaneurysmal SAH are also presented. Medical resources utilization in patients following SAH between the medical center and regional hospital is reported on a nationwide population-based study.

DCI, a result of different pathological pathways, is a complex process and has shown its importance as the leading determinant of poor functional outcome in patients surviving the initial hemorrhagic insult of SAH. The possible mechanisms of EBI and DCI after SAH, as well as their relationship with CV, are illustrated in Figure 1. The importance of CV in DCI has long been overemphasized. CV is not the sole or necessary process leading to DCI. Treatment strategies targeting at CV prevention alone are not adequate. Considering CV as the only monitor of therapeutic effectiveness or the lone prognostic marker can be misleading. Strategies focusing on the detection and treatment of EBI as an alleviation of the occurrence of DCI to subsequently improve overall outcome could make promising future study blueprints.

*Chih-Lung Lin* *Aaron S. Dumont* *John H. Zhang* *Mario Zuccarello* *Carl Muroi*

## Figures and Tables

**Figure 1:**
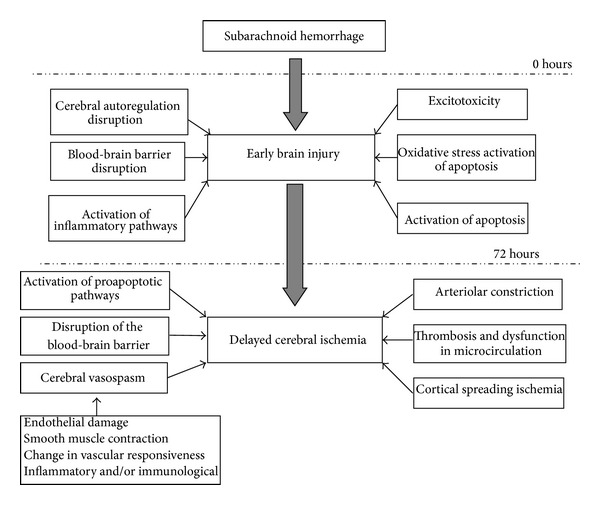
The mechanisms of early brain injury and delayed cerebral ischemia following subarachnoid hemorrhage.
